# The Role of Tumor Markers: Carcinoembryonic Antigen and Cancer Antigen 15-3 in Patients With Breast Cancer

**DOI:** 10.7759/cureus.16298

**Published:** 2021-07-10

**Authors:** Maimoona Khushk, Adil Khan, Abdur Rehman, Sehrish Sheraz, Yar Muhammad Tunio, Kubra Rehman, Duaa Rehman, Moiz Ahmed, Kiran Abbas, Muhammad E Khan

**Affiliations:** 1 Department of Surgery, Peoples Medical College Hospital, Nawabshah, PAK; 2 Department of Surgery, Shifa International Hospital, Islamabad, PAK; 3 Department of Medicine, Usman Memorial Hospital, Karachi, PAK; 4 Department of Public Health, Dow University of Health Sciences, Karachi, PAK; 5 Department of Medicine, Gambat Institute of Medical Science, Khairpur, PAK; 6 Department of Medicine, Shaheed Mohtarma Benazir Bhutto Medical College, Karachi, PAK; 7 Department of Medicine, Liaquat National Hospital and Medical College, Karachi, PAK; 8 Department of Medicine, Jinnah Postgraduate Medical Centre, Karachi, PAK; 9 Department of Medicine and Surgery, Sindh Medical College, Karachi, PAK; 10 Department of Oncology, Punjab Institute of Nuclear Medicine and Radiotherapy, Faisalabad, PAK

**Keywords:** ca 15-3, cea, breast cancer, cancer antigen 15-3, carcinoembryonic antigen

## Abstract

Introduction

Breast cancer is a major cause of mortality among females, worldwide. The present study was intended to evaluate the significance in the management of carcinoembryonic antigen (CEA) and cancer antigen 15-3 (CA15-3) in patients with breast cancer.

Methodology

A cohort study was conducted at the Jinnah Postgraduate Medical Center, Karachi, Pakistan from June 2020 to May 2021. All diagnosed cases of breast cancer who underwent surgical excision of tumor were eligible to partake. Patients who had metastatic breast cancer or had a recurrence were excluded. The patient’s sociodemographic and clinical data were documented in a predefined pro forma. It included information about the age, sex, weight, as well as serum CEA and CA15-3. The CA15-3 and CEA levels for each patient were assessed by taking a 5ml blood sample and sending it to the laboratory for further workup. preoperatively on the second, seventh, and 28th postoperative days.

Results

A mean ± SD age of 52.6 ± 8.89 years was reported. Family history of breast cancer was positive in one-fourth of the patients. Nodal metastasis was negative in 114 (46.72%) patients. Three-fourth of patients had Stage II-IV with only a minority diagnosed with Stage I. The mean levels for CA15-3 in women with Stage I cancer was significantly lower on the seventh day and 28th postoperative day, compared to preoperative levels (p = 0.05). Similar associations were seen for stages II and III. Higher CEA levels were significantly associated with stage III breast cancer preoperatively (5.88 ng/ml, p = 0.05) compared to postoperative values.

Conclusion

The current study revealed that preoperative values of serum CEA and CA15-3 significantly reduced postoperatively. Moreover, patients with advanced cancers had significantly higher levels of both tumor markers than those with less advanced diseases. The current study highlighted the importance of regular assessment of serum CEA and CA15-3 in breast cancer patients. Both these biomarkers are substantially elevated in breast cancer patients, preoperatively. Determining the levels of serum CEA and CA15-3 pre- and postoperatively may determine the prognosis and aid in forming the most optimal patient care regime with respect to the stage and subtype of cancer.

## Introduction

The United States Centers for Disease Control (CDC) has reported breast cancer to be one of the major causes of death among females throughout the world [1]. Breast malignancy is diagnosed in over a million women each year and results in around four hundred thousand deaths [2-5].

In Pakistan, breast carcinomas consist of 14% of all tumors in women, thus are the most prevalent malignancy in the region [6]. in Karachi alone, Pakistan's largest city breast cancer comprises one-third of all tumors [7]. Survival in breast cancer is influenced by prompt diagnosis, appropriate treatment strategies, and genetic susceptibility. On the other hand, the prognosis is largely dependent on the histologic type of tumor, its grade, size, and distant metastasis. However, in many cases due to delayed presentation, lack of awareness, and lack of access to healthcare, by the time diagnosis is established, cancer has advanced to incurable stages.

Therefore, the identification of such tumor markers plays an important role in optimum cancer management. A patient's response to therapy and risk of recurrence can be assessed by regular monitoring of the tumor markers [8]. The use of carcinoembryonic antigen (CEA) and cancer antigen 15-3 (CA15-3) is not extensively explored in our setting. Therefore, the current study evaluated the prognostic role of pre- and post-operative levels of CEA and CA15-3 in the monitoring of disease activity in individuals who had breast cancer. 

## Materials and methods

A cohort study was conducted at the Jinnah Postgraduate Medical Center, Sindh, Pakistan from June 2020 to May 2021, for a duration of 12 months. Ethical approval was obtained from the institute before data acquisition was started. A simple random sampling technique was used to select the patients using Google random number generator. The sample size was calculated using OpenEpi by keeping the likely proportion of 49.5%, a confidence level of 95%, and a margin of error of 6.27%, a sample size of 244 was calculated. Patients were requested for informed written consent prior to participation after the aims and objectives were comprehensively narrated to them. 

All histologically diagnosed cases of breast cancer who underwent surgical excision of tumor were eligible to partake. Patients who had metastatic breast cancer or had a recurrence were excluded. The patient’s sociodemographic and clinical data were documented in a predefined pro forma. It included information about age, sex, weight, as well as serum CEA and CA15-3. The values for both the tumor markers for each patient were assessed preoperatively as well as at the second, seventh, and 28th day, postoperatively.

Statistical Package for the Social Sciences (SPSS version 24; IBM Corp., Armonk, NY) was used to perform data analysis. Chi-square test was applied to assess the correlation between the pre- and post-operative levels of tumor markers. A p-value of <0.05 was set as the cut-off value for statistical significance.

## Results

A mean ± SD age of 52.6 ± 8.89 years was reported. Family history of breast cancer was positive in 59 (24.18%) of the patients. Nodal metastasis was negative in 114 (46.72%) patients. A total of 173 (70.9%) patients had advanced cancer (Stage II and III) with only a minority diagnosed with Stage I. The mean ± SD level of cancer antigen (CA15-3) was 15.7 ± 5.36 U/ml and of CEA was 1.7 ± 1.1 ng/ml. About 86 (35.25%) patients had abnormal levels for CA15-3. Similarly, 45 (18.44%) patients had abnormal CEA levels in our study (Table 1). 

**Table 1:** Clinical characteristics of the 244 patients. CA15-3: cancer antigen 15-3; CEA: carcinoembryonic antigen.

Characteristics	N (%)
Age (mean ± SD)	52.6 ± 8.89 (32-68)
Family history	
Yes	59 (24.18%)
No	185 (75.82%)
Clinical nodal status	
N0	114 (46.72%)
N1	66 (27.05%)
N2	42 (17.21%)
N3 and above	23 (9.43%)
Clinical stage	
I	71 (29.10%)
II	88 (36.07%)
III	85 (34.84%)
Preoperative CA15-3 levels (mean ± SD)	15.7 ± 5.36 (4.0-98)
Normal (<30 U/ml)	158 (64.75%)
Abnormal (>30 U/ml)	86 (35.25%)
Preoperative CEA levels (mean ± SD)	1.7 ± 1.1 (0.6-41)
Normal (<5 ng/ml)	199 (81.56%)
Abnormal (>5 ng/ml)	45 (18.44%)

The mean levels for CA15-3 in patients with Stage I cancer were significantly lower at postoperative seventh day and 28th day, compared to preoperative levels (p = 0.05). Similar associations were seen for stages II and III. Higher CEA levels were significantly associated with stage III breast cancer preoperatively (5.88 ng/ml, p = 0.05) compared to postoperative values (Table 2). 

**Table 2:** Tumor biomarker levels in patients with breast cancer during follow-up, postoperatively. *p = 0.05, **p = 0.01, ***p = 0.001. CA15-3: cancer antigen 15-3; CEA: carcinoembryonic antigen.

Patients (n)	Preoperative	Postoperative (mean, p-value)		
	Mean	2 days	7 days	28 days
CA15-3 in U/mL				
Stage I	18.4	17.21	15.13*	16.88*
Stage II	31.9**	32.43	16.92**	22.33***
Stage III	47.2**	47.11	19.96***	34.98***
CEA in ng/ml				
Stage I	1.95	2.11	2.01	1.69
Stage II	2.69	2.86	2.62	2.22
Stage III	5.88*	5.79	5.71	3.96

It was observed that above normal serum CEA and CA15-3 levels were more frequently reported in patients with advanced tumor stages (Table 3). 

**Table 3:** Frequency of abnormal levels of tumor biomarkers among patients according to the tumor stage. CA15-3: cancer antigen 15-3; CEA: carcinoembryonic antigen.

Tumor biomarker	Stage	Frequency of abnormal levels
CA15-3	Stage I	9.63%
	Stage II	51.06%
	Stage III	39.31%
CEA	Stage I	8.32%
	Stage II	31.30%
	Stage III	60.38%

## Discussion

To monitor the activity of breast cancer tissue, certain biomarkers including CEA and CA15-3 play a vital role. The present study revealed that preoperative values of both aforementioned markers significantly differ postoperatively. Furthermore, patients with advanced cancers had significantly higher levels of CEA and CA15-3 than those with stage I, thus highlighting the importance of these serum markers for monitoring the progression of the tumors. Because the CEA and CA15-3 levels are elevated in patients preoperatively, therefore, they can be used as markers for breast cancer tissue activity. 

Our findings were consistent with a study by Yin et al. who in 2018 identified an association between higher levels of tumor markers CEA and CA-153 in patients suffering from breast cancer [9]. A 2016 study by Fu et al. evaluated the significance of CA15-3 and CEA for establishing the prognosis of breast cancer. The results found a strong correlation between these markers and tumor stage [10]. Another study in 2019 revealed that patients who reported high levels of CA15-3 and CEA had a poorer prognosis compared to those with normal levels of CA15-3 and CEA [11]. Furthermore, the study concluded that the levels of CEA and CA15-3 were considerably associated with the luminal type of breast cancer. 

Taghizadeh et al. in 2019 reported that 65.17% of the patients with breast cancer had elevated levels of serum CEA, while 57.29% of patients had higher CA15-3 levels. The levels of tumor markers had a strong correlation with the advanced stages of cancer [12]. Lian et al. in 2019 revealed similar findings, highlighting the role of tumor markers in breast cancer patients [13]. Based on the existing literature, serum CA15-3 can be utilized as a marker for advanced disease and even metastasis [14]. The preoperative serum levels of CA15-3 and CEA are shown to be elevated in advanced breast cancer disease with greater tumor size, lymph node metastasis, or aggressive histology. This indicates that raised tumor markers are associated with an increased tumor burden [15]. Both oncologists and surgeons are advised to check for serum CA15-3 which can help assess the need for an aggressive treatment protocol [16]. 

The current study had some limitations. Firstly, due to limited and undiversified sample size, the findings of the study cannot be generalized to a larger population. Further multi-center studies are needed to ascertain the role of these biomarkers as testing may increase overall healthcare expenses. Secondly, the follow-up period was very short hence, the long-term role of the CEA and CA15-3 should be explored in future studies.

## Conclusions

The current study revealed that preoperative values of serum CEA and CA15-3 significantly reduced postoperatively. Moreover, patients with advanced cancers had significantly higher levels of both tumor markers than those with less advanced diseases. The current study highlighted the importance of regular assessment of serum CEA and CA15-3 - both are substantially elevated in breast cancer patients, preoperatively. Determining the levels of serum CEA and CA15-3 pre- and postoperatively may determine the prognosis and aid in forming the most optimal patient care regime with respect to the stage of cancer.
